# Framing of Poly(arylene-ethynylene) around Carbon Nanotubes and Iodine Doping for the Electrochemical Detection of Dopamine

**DOI:** 10.3390/bios13030308

**Published:** 2023-02-22

**Authors:** Jose Paul, Md Moniruzzaman, Jongsung Kim

**Affiliations:** Department of Chemical and Biological Engineering, Gachon University, 1342 Seongnamdaero, Seongnam-si 13120, Republic of Korea

**Keywords:** poly(arylene-ethynylene)-based COF, CMP-CNT-4 nanocomposite, I-doping, electrochemical sensor, dopamine detection

## Abstract

Dopamine (DA), an organic biomolecule that acts as both a hormone and a neurotransmitter, is essential in regulating emotions and metabolism in living organisms. The accurate determination of DA is important because it indicates early signs of serious neurological disorders. Covalent organic frameworks (COFs) and metal–organic frameworks (MOFs) have received considerable attention in recent years as promising porous materials with an unrivaled degree of tunability for electrochemical biosensing applications. This study adopted a solvothermal strategy for the synthesis of a conjugated microporous poly(arylene ethynylene)-4 (CMP-4) network using the Sonagashira–Hagihara cross-coupling reaction. To increase the crystallinity and electrical conductivity of the material, CMP-4 was enveloped around carbon nanotubes (CNTs), followed by iodine doping. When used as an electrochemical probe, the as-synthesized material (I_2_-CMP-CNT-4) exhibited excellent selectivity and sensitivity to dopamine in the phosphate-buffered solution. The detection limits of the electrochemical sensor were 1 and 1.7 μM based on cyclic voltammetry (CV) and differential pulse voltammetry (DPV).

## 1. Introduction

Dopamine (DA) is a neurotransmitter consisting of a catechol moiety (a benzene ring with two hydroxyl groups at the ortho-position), with one amine group attached via an ethyl chain. In 1958, Carlsson et al. discovered the neurological messenger function of DA, which generates impulses between neurons [1,2]. As a significant element in the human nervous system, DA is necessary for maintaining the neurological health of an individual. Thus, the accurate and selective sensing of DA is essential for physiological purposes. There are several enzyme–antibody aptamer-based fluorescent biosensors and electrochemical methods such as cyclic voltammetry (CV), differential pulse voltammetry (DPV), and aptamer-based colorimetric biosensors are popular for detecting DA because of their quick response and high selectivity [3,4,5,6,7,8,9,10]. The electrochemical detection of DA is favored at pH 7 because it becomes positively charged and can be easily oxidized [11,12]. Recently, multiphoton microscopy has been used to image DA with excitation at 550 nm [13,14,15]. However, uric acid (UA) and ascorbic acid (AA) usually interfere with the fluorescence and electrochemical detection of DA, owing to their structural similarities. Therefore, specific ligands and tuned frameworks are required to selectively distinguish and sense DA from its interferents and analogs.

In this scenario, electrochemical biosensors have been broadly investigated for the sensing and monitoring of neurotransmitters, owing to their high selectivity as well as the possibility of achieving real-time measurements [16]. For this purpose, electrodes made of noble metals (typically platinum or gold), carbon materials, metal oxides, metal–organic frameworks (MOFs), covalent organic frameworks (COFs), and porous organic polymers (POPs) are used to perform local measurements, improve signal-to-noise ratios, and have fast response times [17,18,19,20,21,22,23,24].

MOFs are promising sensing agents utilized in glassy carbon electrodes (GCE) for electrochemical sensing because of their crystallinity, large surface area, and customizable functional sites [25]. Although electrochemical sensing using MOFs is successful to some extent, sensing using COFs and POPs is still in its infancy. When compared with MOFs, the weak crystalline nature of COFs and POPs hinders unidirectional charge mobility and decreases charge transfer. In contrast, the complete organic moiety induces a π-π interaction towards the catechol ring, providing a phase for DA sensing. Hence, crystallinity has a decisive influence on the synthesis of conductive COF. Strong electrophilic and nucleophilic ligands trigger simultaneous bond formation to prepare a crystalline organic polymer with few structural defects. In addition, p-type doping (I_2_) served as a charge carrier and improved the electrical conductivity of the composite material. In conclusion, pre- and post-synthetic modifications, the ability to incorporate conductive materials, host guest self-assembly, and facile syntheses have exposed new possibilities in this field.

Spatial confinement and facile syntheses have motivated the preparation of COFs and POPs for the electrochemical sensing of DA [26,27]. Many POPs contain mobile π-electrons, but their lack of crystallinity and good charge carriers limit their electrical conductivity [28,29,30,31,32]. In such cases, the enveloping of POPs around carbon nanotubes (CNTs) provides a tubular organic crystalline framework that can boost charge transfer [32,33]. CNTs are generally used as electrode modifiers in biosensing, and their conductive properties depend on the orientation of the graphene lattice with respect to the tube axis. COF-functionalized CNTs exhibit good electrocatalytic properties for the oxidation and reduction in DA, owing to their enhanced electrical conductivity, large specific surface area, and good mechanical and chemical stability [33,34,35].

**Scheme 1:**
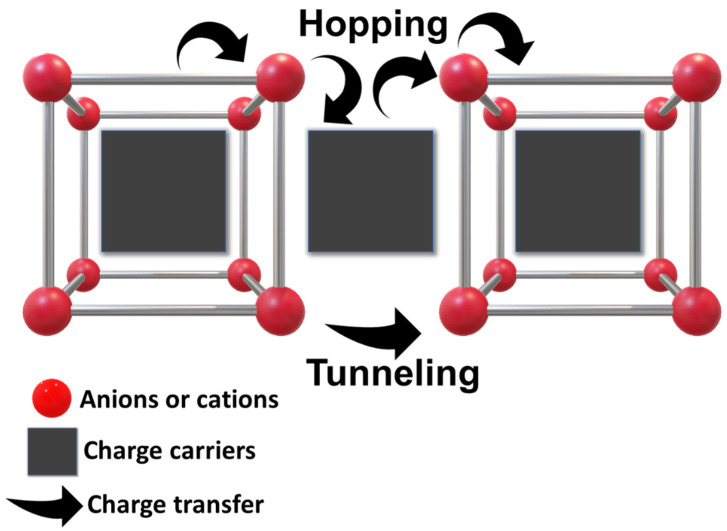
Hopping and tunneling mode of charge transfer mechanism in SSOPMs.

A crucial objective in the synthesis and modification of COFs is to obtain highly conjugated, charge-carrying, and crystalline structures with minimal defects [36,37,38,39,40]. An ordered crystalline framework enhances unidirectional π-electron mobility in the COF [41,42]. Charge delocalization depends on the orbital overlap of transporters and carriers in the building blocks. Hopping and ballistic (or band-like) (Scheme 1) are the two main charge transfer mechanisms in solid-state-ordered porous materials (SSOPMs). The mechanism of charge transfer in hopping mode is associated with electron or hole hopping between unbonded neighboring sites where the charge carriers are localized. Meanwhile, there is a continuous energy band between the adjacent units for ballistic transport. In brief, there should be an orbital overlap between the electron transporters and carriers in the building blocks, which is necessary for charge delocalization.

**Scheme 2:**
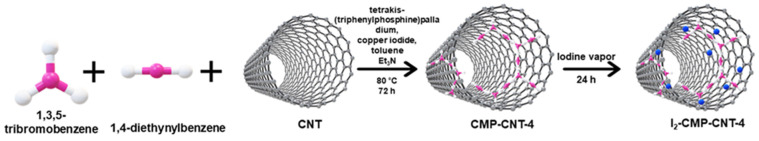
Synthesis of the I_2_-CMP-CNT-4 nanocomposite.

**Scheme 3:**
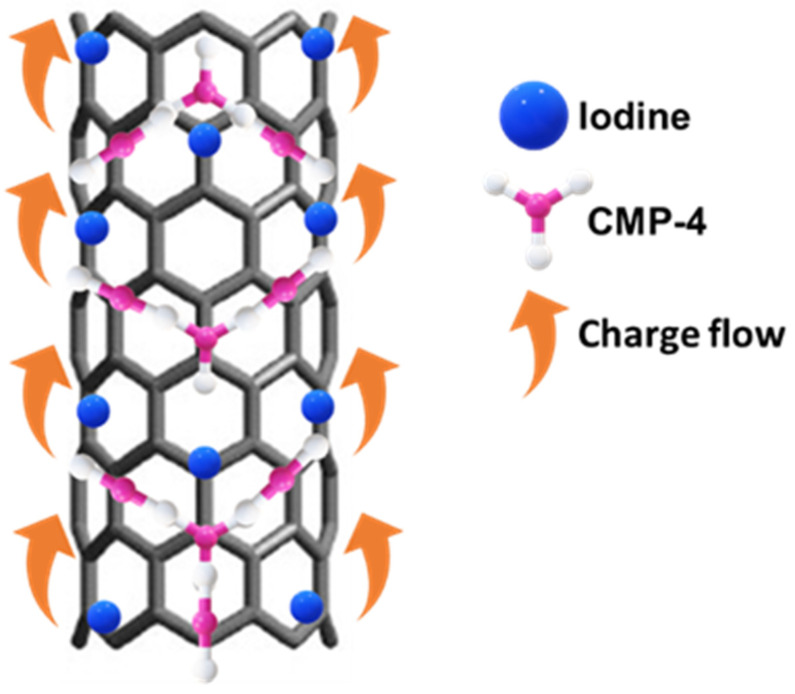
Hopping mode of the charge transfer mechanism in the I_2_-CMP-CNT-4 nanocomposite.

Based on the preceding characteristics, we synthesized a COF-CNT composite of a conjugated microporous poly(arylene-ethynylene)-4 network (CMP-CNT-4) through the Sonagashira–Hagihara cross-coupling reaction [27]. The enveloping of CMP-4 around the CNT provides a tubular organic crystalline framework that can enhance unidirectional charge delocalization (Scheme 2). The doping of I_2_ into CMP-CNT-4 boosted the electrochemical sensitivity of the entire composite towards DA through the hopping charge transfer mechanism (Scheme 3). The morphological, optical, and chemical properties of the I_2_-CMP-CNT-4 nanocomposite were investigated using various characterization techniques, such as X-ray diffraction (XRD), scanning electron microscopy (SEM), transmission electron microscopy (TEM), Fourier transform infrared (FT-IR) spectroscopy, ultraviolet-visible (UV–Vis) absorption spectroscopy, Raman spectroscopy, X-ray photoelectron spectroscopy (XPS), and nuclear magnetic resonance (NMR) spectroscopy. Voltammetry investigations were performed using a WizMac WizEIS-1200 Premium voltammeter.

## 2. Experimental Section

Herein, we utilized a solvothermal method for the synthesis of brown-colored CMP-4 and CMP-CNT-4 networks by reacting 1,4-diethynylbenzene and 1,3,5-tribromobenzene-based linkers in toluene as the solvent (Figure S1, ESI†). The wrapping of CMP-4 around the CNT was conducted using a minor modification of a previously reported method by dispersing CNTs into the reaction mixture, as shown in the electronic Supplementary Information. In addition, the gravimetric iodine adsorption method was used to dope I_2_ vapors on the CMP-CNT-4 surface.

All chemicals and solvents were purchased from commercial suppliers and used without further purification. 1,4-diethynylbenzene [C_10_H_6_], 1,3,5-tribromobenzene [C_6_H_3_Br_3_], tetrakis-triphenylphosphine)palladium [C_72_H_60_P_4_Pd], copper iodide [CuI], toluene [C_7_H_8_], triethylamine [Et_3_N], chloroform [CHCl_3_], dopamine hydrochloride [C_8_H_11_NO_2_·HCl], L-cysteine, glutamic acid, arginine, ascorbic acid [C_6_H_8_O_6_], zinc sulfate heptahydrate [ZnSO_4_·7H_2_O], and zinc acetate dihydrate [Zn(CH₃CO₂)₂·2H₂O], sodium chloride, potassium chloride, calcium chloride, magnesium nitrate, sodium sulfate, methanol, acetone, carbon nanotube, and iodine were obtained from Sigma Aldrich. Hydrochloric acid (HCl) and ethanol were obtained from Duksan (Ansan, Republic of Korea) Fetal bovine serum (FBS) was obtained from Gibco. Deionized water and phosphate-buffered saline (PBS) (10 mM, pH 7.4) were used for the sensing experiments.

Solid-state 13C and 1H NMR spectra were recorded on a Bruker Advance III HD (Bruker, Germany) 500 MHz spectrometer; chemical shifts are represented in δ (ppm units). Powder X-ray diffraction (PXRD) patterns of the samples were measured using a Smart Lab instrument (Rigaku) with D/teX Ultra 250 detectors under a 4-kW X-ray generator (scan range: θ/2θ = 5–80°). Fourier transform infrared spectra were recorded in the range of 200–4000 cm^−1^ on Vertex 70 (Bruker, Billerica, MA, USA). Powder samples were dried, and subsequently, a small amount of the powder was placed directly between two support plates without hygroscopic materials (such as NaCl or KBr). SEM images were acquired from a Hitachi S-4700 FE-SEM (Japan). For all samples, imaging was performed in a high vacuum at an accelerating voltage of 15 kV, working distance of 11.7 mm, ×2.00 k magnification, and an emission current of 9000 nA. TEM specimens were prepared by placing one drop (10 μL) of the CMP and CNT-CMP ethanol-dispersed solution onto a carbon-coated copper grid and drying it in an oven for 6 h at 60 °C. All the sample solutions were treated in an ultrasonic bath (15 min) before the deposition to reduce aggregation. TEM was performed using JEM 3010, JEOL Ltd., Japan, at 300 kV, with a total magnification of ×22.00 k and indicated magnification of ×29.00 k. UV–Vis absorption spectra were recorded on a (Varian Cary 100) spectrophotometer using ethanol as the solvent. CV, DPV, and EIS were performed using a WizMac WizEIS-1200 Premium voltammeter at room temperature in a standard three-electrode cell, where the working electrode was glassy carbon (GC) (area = 0.07 cm^2^), the counter electrode was a Pt wire, and the Ag/AgCl/KCl (sat) electrode was used as the reference electrode.

The glassy calomel electrode was thoroughly washed with acetone, ethanol, and double distilled water, and polished by using alumina before drop casting. For casting, 0.5 mg I_2_-CMP-CNT-4 was dispersed in 0.5 mL ethanol followed by sonication for 20 min. A drop (20 μL) of well-dispersed composite material in the liquid-dispersed phase was added to the center of the glassy calomel electrode and the casted electrode was allowed to dry at room temperature. Likewise, CV comparisons for modified GCE, scan rate, the Nyquist plot, CV and DPV plot for various concentrations of DA, stability, reproducibility, and interference in PBS and FBS were conducted at 7.4 pH, respectively. For the EIS measurement of bare and modified GCE, we applied the frequency range from 0.1 to 10^5^ Hz in PBS (7.4 pH). Since the pH of human blood is around 7.35–7.45, 7.4 was selected for various electrochemical measurements. Additionally, we studied the pH response in 7.62, 7.27, and 6.27 by adding dilute hydrochloric acid to the analyte solution.

### 2.1. Preparation of CMP-4

1,4-diethynylbenzene (50 mg, 0.396 mmol) and 1,3,5-tribromobenzene (49.8 mg, 0.158 mmol) were dissolved in a mixture of toluene (5 mL) and Et_3_N (2.5 mL) in a 50 mL glass vial. Tetrakis(triphenylphosphine)palladium (11 mg) and copper iodide (3.5 mg) were added to the brown-colored solution and purged with nitrogen gas to prevent the homocoupling of alkyne monomers. The reaction mixture was heated to 80 °C for 72 h. Subsequently, the mixture was cooled to room temperature step-by-step. The precipitated polymer was filtered through a 90 mm filter paper and washed four times with chloroform, water, methanol, and acetone. CMP-4 polymer (Figure S2a) was purified by Soxhlet extraction from methanol for 48 h. The product dried in an oven for 6 h at 60 °C.

### 2.2. Preparation of CMP-CNT-4

We followed the same procedure in Section 2.1 for the synthesis of CMP-CNT-4. An extra 10 mg of CNTs was added to the brown-colored solution containing 1,4-diethynylbenzene and 1,3,5-tribromobenzene. Tetrakis(triphenylphosphine)palladium (11 mg) and copper iodide (3.5 mg) were added to the solution, which was purged with nitrogen gas. The reaction mixture was heated to 80 °C for 72 h. The precipitated polymer network was filtered and washed four times with chloroform, water, methanol, and acetone. The Soxhlet extraction (Figure S2b) using methanol was used for further purification.

### 2.3. Synthesis of I_2_-CMP-CNT-4

We adopted a gravimetric method for the adsorption of iodine onto CMP-CNT-4. A vial (1.5 mL) containing 20 mg of dried CMP-CNT-4 powder was placed into a 50 mL vial, which was charged with 500 mg of iodine. Then, it was capped, heated to 35 °C, and kept in dark for 24 h, yielding black-colored iodine adsorbed I_2_-CMP-CNT-4.

## 3. Results and Discussion

### 3.1. Physical and Chemical Characterization of the CMP-4 and CMP-CNT-4

**Figure 1:**
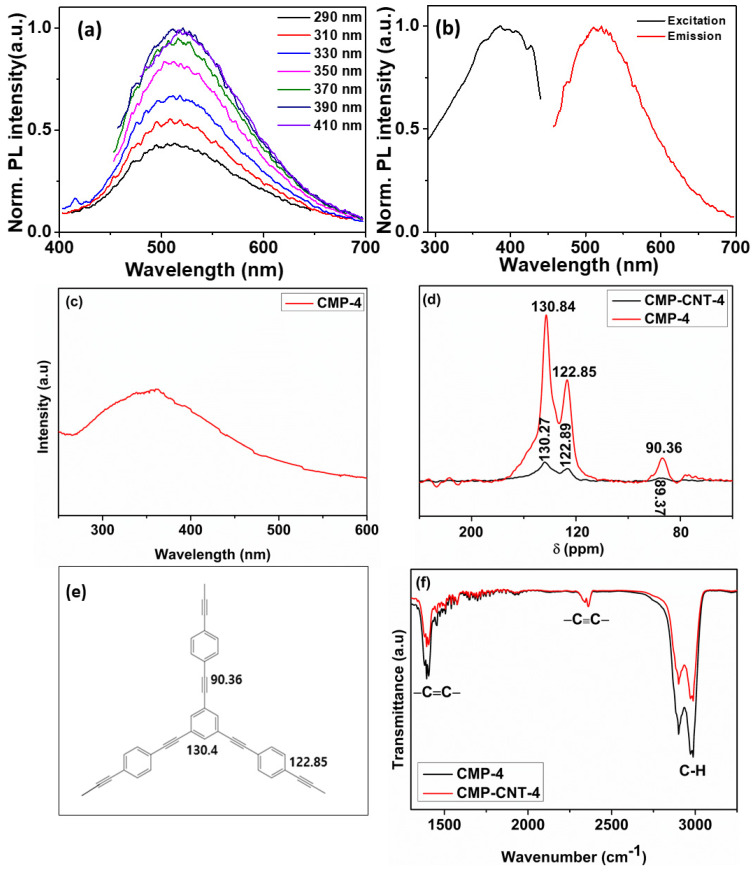
(**a**) Wavelength-depended fluorescence emission spectra of CMP-4; (**b**) excitation and emission maximum spectra of CMP-4; (**c**) UV–Vis spectra of CMP-4; (**d**) solid-state C^13^ NMR spectra of CMP-4 and CMP-CNT-4; (**e**) structure and ^13^C NMR value for CMP-4 and the orientation of π electrons. (**f**) FT-IR spectrum of CMP-4 and CMP-CNT-4.

The optical properties of the as-synthesized COF (CMP-4) were determined using photoluminescence (PL) emission spectra. The alkyne-linked polymer exhibited a bright yellowish-green fluorescence under UV illumination (Figure S2e, ESI†). Figure 1a shows photoexcitation at various wavelengths ranging from 290 to 410 nm. Poly(arylene-ethynylene) exhibited almost excitation-independent emission properties. The emission maximum (λmax) was 510 nm (Figure 1b) under excitation at 390 nm, owing to the π–π* transition of the conjugated π-electrons perpendicular to the molecular plane. Fluorescence is generally observed in the lowest energy transition, which is π* → π for the delocalized electrons in poly(arylene-ethylene). Figure 1c shows the UV–Vis absorption spectrum of the solid poly(arylene-ethynylene) sample dispersed in ethanol, which has a peak at 360 nm.

The carbon atoms in poly(arylene-ethynylene) are in three different chemical environments (Figure 1d). Two highly de-shielded carbons in the aromatic ring and one triply bound, shielded alkynyl carbon outside the aromatic ring gave three distinct peaks in 13C-NMR spectra. The triply bonded carbon exhibited shifts of 89.37 and 90.36 ppm for CMP-4 and CMP-CNT-4, respectively, while the acetylene-bonded carbon within the aromatic ring showed shifts of 122.89 and 122.85 ppm. The un-bonded carbon had shifts of 130.24 and 130.84 ppm. Figure 1e shows the chemical structure of poly(arylene-ethynylene), annotated with 13C NMR shifts in ppm.

The chemical configuration of the synthesized poly(arylene-ethynylene) was identified using three diagnostic FT-IR peaks (Figure 1f). Disubstituted –C≡C– stretching, aromatic –C=C– bending, and aromatic C–H stretching resulted in transmittance peaks at 2360, 1394, and 3030 cm^−1^, respectively.

In conclusion, the synthesis of CMP-4 and CMP-CNT-4 was confirmed through physical and chemical analyses.

### 3.2. Morphological Characterization of the CMP-CNT-4

**Figure 2:**
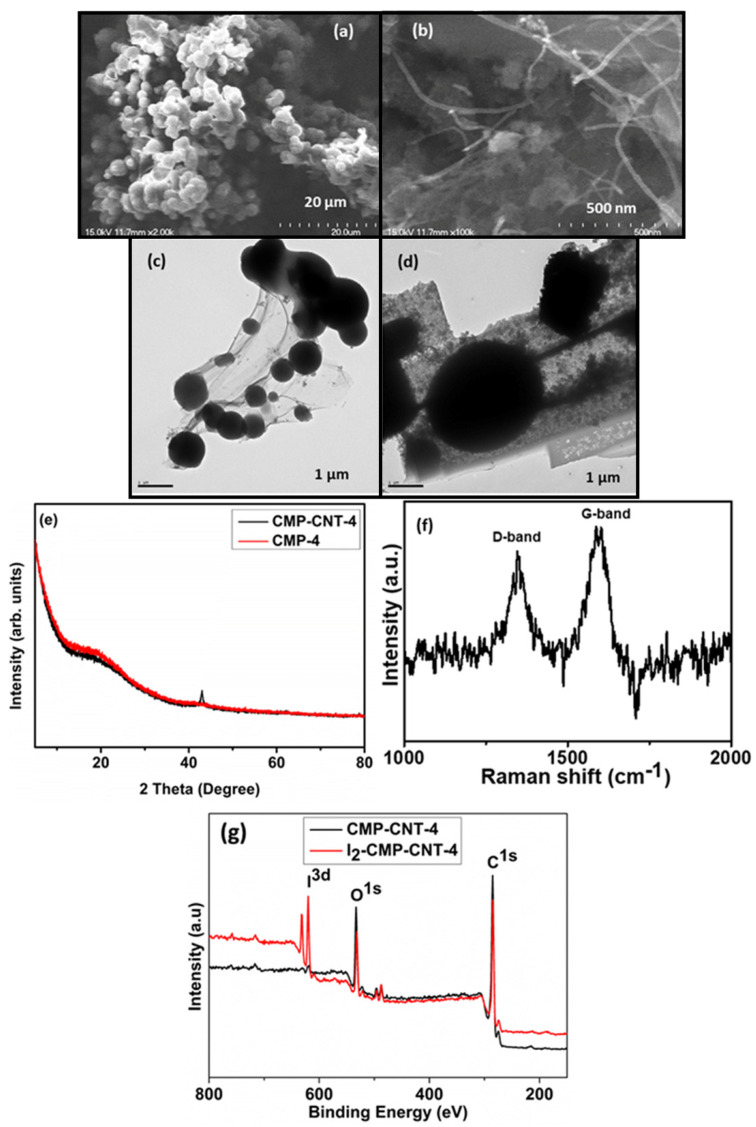
(**a**) SEM image of CMP-4, (**b**) SEM image of CMP-CNT-4, (**c**) TEM images of CMP-4 (**d**) TEM image of CMP-CNT-4, (**e**) XRD patterns of CMP-4 and CMP-CNT-4, (**f**) Raman spectra of CMP-CNT-4, (**g**) XPS spectra of CMP-CNT-4 and I_2_-CMP-CNT-4.

SEM was used to assess the surface structure and shape of as-synthesized CMP-4 and CMP-CNT-4 (Figure 2a,b and Figure S3 (ESI†)). Analysis of the brown-colored CMP-4 revealed that the material was composed of solid spheres (with sub-micrometer dimensions), as shown in Figure 2a. The SEM image of the CMP-CNT-4 (Figure 2b and Figure S4b) nanocomposite exhibits tubular structures along with spheres, indicating the successful wrapping of CMP-4 around the CNT [27]. TEM was employed to evaluate the external morphology and imperfections of CMP-4 and CMP-CNT-4, as shown in Figure 2c,d. The TEM images were in accordance with the SEM analysis and proved that the spherical CMP-4 particles had an average size of 1 μm (Figure S4a, ESI†). These spheres were also found to be wrapped over the CNT, yielding tubular CMP-CNT-4 structures (Figure 2d) [27]. Physical and morphological analyses confirmed the presence of CNT nanowires in the CMP-4 framework, confirming that CMP-CNT-4 was a nanocomposite.

XRD was employed to inspect the crystal phase and defect nature of the CMP-4 and CMP-CNT-4 powders. XRD patterns (Figure 2e) from 5–80° exhibited a broad diffraction peak around θ = 20°, indicating that CMP-4 is an extremely weak crystalline in nature. CMP-4 was prepared under kinetically driven conditions, which gave an amorphous morphology. However, owing to the wrapping around CNT, the CMP-CNT-4 composite showed a diffraction peak near 40°, which corresponds to the (100) plane of carbon from CNT. The XRD data confirmed that the peak detected from the composite is from the CNT-induced crystallization of CMP-4.

Raman spectroscopy was performed to further analyze the intrinsic structure of CMP-CNT-4. As shown in Figure 2f, the peak at 1350 cm^−1^ corresponds to the D-band, which suggests the presence of disordered carbon frameworks, while the peak at 1590 cm^−1^ represents the G-band, indicating the presence of sp^2^ hybridized carbon in the framework. As the crystalline nature provides a smooth Raman plot for CNT (Figure S6), the noise in the CMP-CNT-4 plot is due to the weak crystalline nature of CMP-4 (Figure 2f). Similarly, the presence of sp^3^ and sp^2^ carbon in the CNT gives the D and G bands in the Raman spectra. The absence of the sp^3^ carbon in CMP-4 gives only the G band in the Raman spectra (Figure S6). Overall, the intense G band compared to the D band in the composite is from the resultant peak of CNT and CMP-4 (Figure S6).

The XPS survey spectrum (Figure 2g and Figure S23) revealed the presence of C(1s) and I (3p, 3d, and 4d) core–shell electronic energy levels in the I_2_-CMP-CNT-4. C(1s) (Figure S23) in CMP-CNT-4 were in three different chemical environments (1 sp and 2 sp^2^) exhibiting a peak at 283.9, 285.4, and 287.1 eV, respectively. To be clear, carbon from the outer layer in CMP-CNT-4 showed an XPS peak for this composite. Moreover, the C(1s) peak was unresponsive after doping, confirming the physical interaction between the carbon in CMP-4 and adsorbed I_2_. In general, alkynyl bonding provided chemical stability to the CMP-CNT-4 network.

The permanent porosity of the CMP-CNT-4 network was examined by gas sorption measurements using nitrogen at 77 K after activation at 100 °C for 8 h (Figure S7a–c). The Brunauer–Emmett–Teller (BET) surface area of CMP-CNT-4 was calculated as 998.712 m^2^/g. In isotherm studies, the improved nitrogen uptake above a relative pressure of 0.2 and the increase in uptake above P/P_0_ of 0.8 is due to the combination of the tubular and microporous nature of the CMP-CNT-4 network.

In summary, we synthesized I_2_-CMP-CNT-4 with a microporous size and rigid monomer, owing to its alkynyl bonding.

### 3.3. Electrochemical Behavior of a Modified GCE

**Figure 3:**
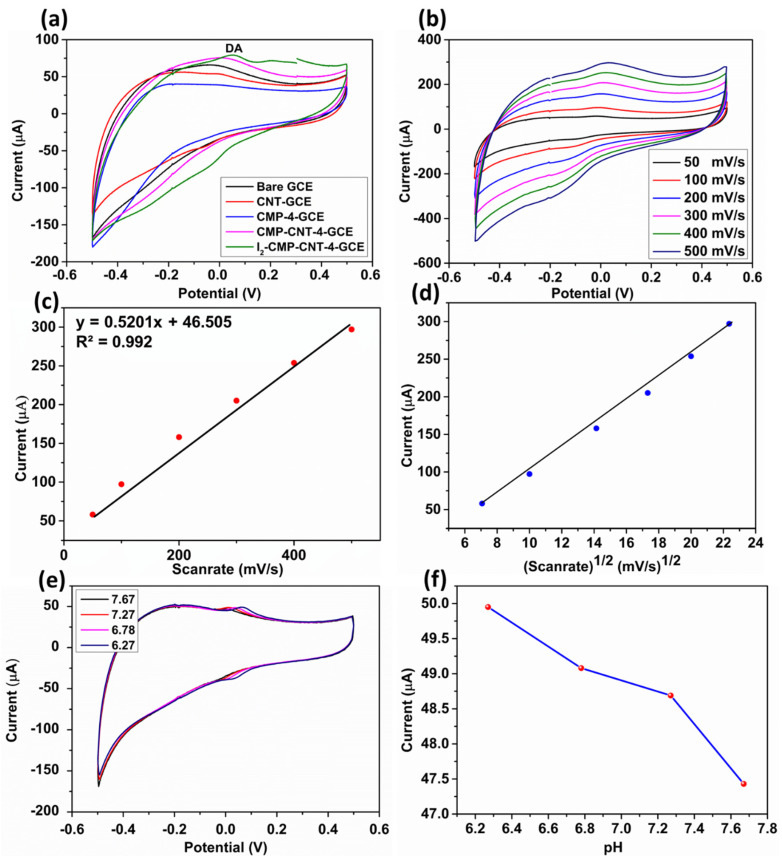
(**a**) CV curves of the bare GCE, CNT-modified GCE, CMP-4-modified GCE, CMP-CNT-4-modified GCE, and I_2_-CMP-CNT-4-modified GCE in 4 μM DA, (**b**) CV plot for peak current vs. scan rate from 50 to 500 mV/s in 4 μM DA, (**c**) linear regression plot for peak current vs. scan rate from 50–500 mV/s, (**d**) linear plot for peak current vs. (scan rate)^1/2^ from 50–500 mV/s, (**e**) CV plot for 1 μM DA at different pH, and (**f**) linear plot for peak current vs. pH in 1 μM DA at different pH.

Figure 3a displays the electrochemical response of the modified electrodes in 4 μM DA in 0.1 M PBS at pH 7.4. The peak current (I) of the CNT-modified GCE was higher than that of the bare GCE, indicating facile electron transfer (S21). The current intensity (75.27 μA) of the CMP-CNT-4 modified GCE showed a sharp increase compared to that of the CMP-4 electrode. This was because of the improved composite formation and cooperation, which facilitated electron transfer and increased the electrochemically active surface area (EASA). The electrode modified by I_2_-doped CMP-CNT-4 showed amplified anodic and cathodic current peaks (79.04 μA) toward the redox reaction due to the adsorption of I_2_ on the CMP-CNT-4 surface. This increased the electron transfer ability and EASA of the material because the lone pair of electrons in the valence shell of I_2_ could act as a charge carrier.

The scan rate, which shows the pace of fluctuation of the electric potential with respect to time, is a decisive parameter in CV experiments. A study of the redox behavior of 4 μM DA in 0.1 M PBS (7.4 pH) by sweeping the voltage from 50 to 500 mV/s was performed for the I_2_-CMP-CNT-4-modified GCE. The results demonstrated that a high scan rate increased both the oxidation and reduction peak currents (Figure 3b) and caused a slight shift in the redox peak potential, owing to the smaller diffusion layer [43,44]. The kinetic behavior of the nanocomposite was calculated using the mathematical relationship between the voltammetry current response of the composite material and the scan rate. A calibration curve (Figure 3c) was plotted, and the current response exhibited a linear relationship (R^2^ = 0.989) for scan rates in the range of 100–500 mV. Figure 3d shows the current vs. (scan rate)^1/2^ for the diffusion mechanism. The linear plot in Figure 3c,d confirms that this process has diffusion- and adsorption-controlled mechanisms.

Since DA adopts a diffusion and adsorption-controlled mechanism towards the I_2_-CMP-CNT-4-modified GCE. This mixed kinetic mechanism is proved through ‘Ip’ vs. ‘scan rate’ and ‘Ip’ vs. (scan rate)^1/2^ linear plot. In this context, the log(A) vs. log(V) plot further confirms the exact sensing mechanism through its slope (Figure S10). It is well known that a slope near 1 is for adsorption controlled, and near 0.5 is for a diffusion-controlled process [35]. Figure S10 shows the relationship between log A and log V (scan rate), which follows the linear equation y = 0.6825x + 0.6325 (log(A) = 0.6825log(V) + 0.6325). Based on the log(A) vs. log(V) plot, where the slope is 0.68, it is evident that diffusion dominates over adsorption whereas, to some extent, it still shows simultaneous adsorption- and diffusion-controlled kinetics.

The Nyquist plots (Figure S11, ESI†) are the resultant Faradaic impedance spectrum, which provides information on parameters such as double-layer capacitance, Warburg diffusional impedance, and ohmic resistance [42]. It was observed that the I_2_-CMP-CNT-4, CMP-CNT-4, and CMP-4-modified GCE showed lower Ret values than the bare GCE. Interestingly, after modification of the GCE surface with CMP-4, CMP-CNT-4, and I_2_-CMP-CNT-4, the diameter of the semicircle portion of the Nyquist plot gradually decreased, which implies a gradual increase in conductivity and faster electron transfer. The tubular network of CNT and the enveloped CMP-4 enhanced the unidirectional charge mobility. Iodine serves as the charge carrier, accepting and donating electrons throughout the CMP-CNT composite.

pH is a vital parameter that significantly affects the electrochemical performance of the modified GCE. To monitor the influence of protons in an electrochemical redox reaction, the peak response towards various pH values 6.27 to 7.67 was performed in 0.1 M PBS and 4 μM DA. Figure 3d shows that the Ip of DA increased for pH values up to 5. This is due to the π-π interactions between CMP-4 and DA, as well as the adsorption of protonated DA to the CMP-CNT-4 surfaces through the lone pair of I_2_. Conversely, because the electrochemical oxidation of DA includes deprotonation, the reaction is weaker in extremely acidic environments, which leads to a drop in the peak currents [45,46].

### 3.4. Electrochemical Detection of DA and Sensing Mechanism

**Figure 4:**
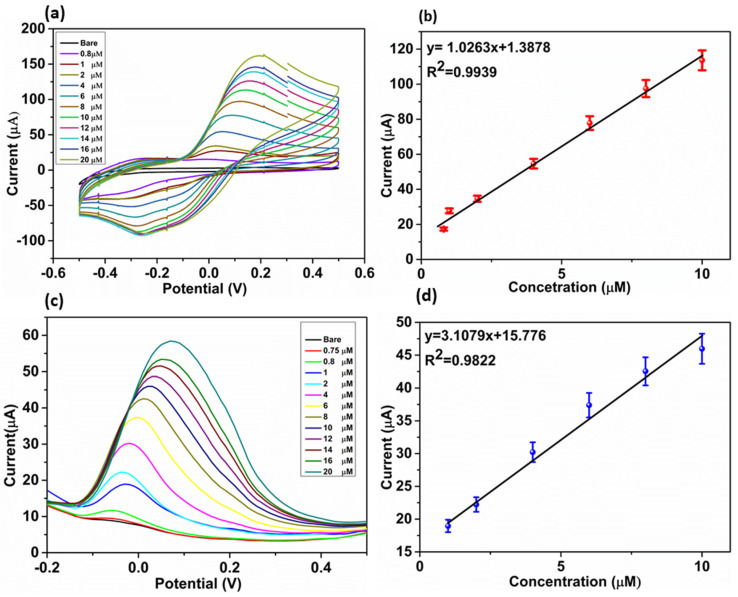
(**a**) CV curves of I_2_-CMP-CNT-4 with the following addition of DA (0.8 to 20 µM); (**b**) plot of anodic peak current vs. concentration (0.8–10 µmolar DA); (**c**) DPV curves of I_2_-CNT-CMP-4 with following the addition of DA (0.75–20 µM); (**d**) plot of oxidation peak current vs. concentration (1–10 µM DA).

The I_2_-CMP-CNT-4-modified GCE exhibited a strong reversible peak towards DA, and the current increased gradually from 17 to 160 μA with the addition of 0.8−20 μM DA in the CV measurements (Figure 4a). To formulate the mathematical relationship between the voltammetric current response of the composite material and DA concentration, a calibration curve (Figure 4b) was plotted. The redox response showed a linear relationship with DA concentration in the range of 0.8–10 μM. CV studies clearly showed that the peak current response is highly sensitive and has good linearity with DA concentration in the range of 0.8–10 μM (R^2^ = 0.9939); the limit of detection (*LOD*) was determined to be 1 μM. *LOD* was calculated based on the standard deviation of the result (*Sy*) and the slope of the calibration curve (*S*). *LOD* according to the formula is;

(1) $$LOD=3.3\frac{Sy}{S}$$

Therefore, the remarkable current response of the nanocomposite compared to that of the bare GCE can be explained by its larger surface area, more favorable electron transfer, increased ability to adsorb biomolecules, and favorable environment for dopamine oxidation [43].

A more sensitive technique called DPV was applied to monitor DA oxidation, which resulted in an anodic wave at a voltage near −0.05 V. The best potential step value was derived using a pulse width of 50 ms, pulse amplitude of 20 mV, step period of 0.1 s, and step height of 100 mV in the potential range from −0.5 to +0.5 V. Quantitative measurements were performed for DA in the concentration range from 0.75 to 20 μM (Figure 4c), which shows a gradual increase in the current from 9.64 to 60 μA. As shown in Figure 4c, the peak position changed throughout the experiment, indicating that the surface was poisoned during each experimental cycle. This is due to the passivation of the electrode surface by the synthesis of melanin from DA and dopamine chrome. Melanin forms an impermeable layer that inhibits direct contact between the analyte and the electrode surface. To formulate the mathematical relationship between the voltammetric current response of the composite material and the DA concentration, a calibration curve (Figure 4d) was plotted, and the current response showed a linear relationship with the DA concentration in the range of 1–10 μM. DPV studies clearly showed that the peak current response had good linearity with DA concentrations in the range of 1–10 μM (R^2^ = 0.9822); the limit of detection was determined to be 1.7 μM. Calculations based on these results suggest that the electrode reactions proceed through a two-electron transfer with the release of two protons [45]. In cyclic voltammetry measurements, the redox reaction was observed to be diffusion-controlled; however, the DPV data indicated that DA oxidation was preceded by the adsorption of molecules onto the electrode surface [46,47,48,49,50]. Table 1 presents a comparison of the I_2_-CMP-CNT-4-modified GCE with the other modified GCEs, indicating the superiority of our material.

The DPV response in FBS was recorded for the real sample response of the I_2_-CMP-CNT-4-modified GCE towards DA (Figure S12). For this study, 20 mL of FBS was directly added to 0.1M PBS (7.4 pH), and this sample was further spiked with a known concentration of DA (8–18 μM). There was an increase in the peak current response of the FBS-PBS solution according to the DA concentration, as shown in Figure S12. Moreover, a calibration curve for ‘Current Vs. Concentration’ (Figure S12) was plotted, where the current response showed a linear relationship with DA concentration in the range of 8–18 μM. Thus, the DPV studies clearly show that the peak current response has good linearity with DA concentrations in the range of 8–18 μM (R^2^ = 0.9924).

### 3.5. Interference, Stability, and Reproducibility Study

**Figure 5:**
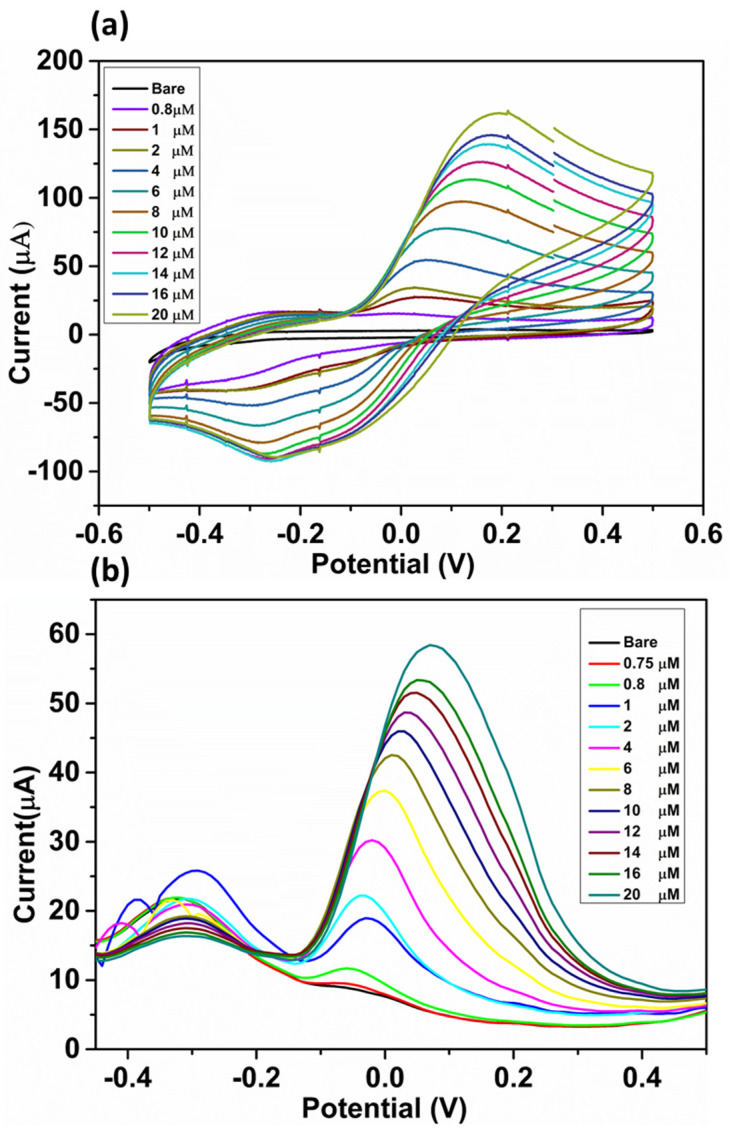
(**a**) CV plot for the interference study of AA and DA (1–20 µM), (**b**) DPV plot for the interference study of DA and AA (1–20 µM).

Analogs and interferents curtail the detection of DA by voltammetry [59]. The DPV plot (Figure S13) proves the insensitive nature of the I_2_-CMP-CNT-4-modified GCE towards 1 μM AA in PBS (pH 7.4). Simultaneous sensing of DA and AA was investigated through CV and DPV in the concentration range of 1–20 μM in a 0.1 M phosphate-buffered electrolyte solution. Figure 5a,b show the CV and DPV signals for DA in the presence of interfering ascorbic acid (AA). As shown in Figure 5a, the CV investigation reveals the insensitive nature of the I_2_-CMP-CNT-4-modified GCE towards AA. In the DPV measurement (Figure 5b), the I_2_-CMP-CNT-4 modified GCE exhibits two distinct peak currents (−0.3 & 0.00 V) due to AA and DA oxidation, respectively. Similarly, the interference of the modified GCE towards other anions such as sulfate, nitrate, acetate, and urea (Ur) was evaluated using CV and DPV in the presence of 1 μM DA. Figure S14a–e again confirms the insensitive nature of the I_2_-CMP-CNT-4-modified GCE. Additionally, Figure S15 displays the DPV plots of DA (1 μM), L-cysteine (L-Cyst, 1 μM), and glutamic acid (GA, 1 μM). From the DPV plot, we can observe that the oxidation peak current position of DA is distinguishable by the addition of amino acids to the PBS. However, cationic interferents (Figure S16), such as Ca^2+^, K^+^, and Na^+^ (individual concentration of 1 μM), enhanced the peak current value of the modified electrode in the DPV analysis for DA.

The response to various interferents such as analog (AA), amino acid (Cyst, Argrn), cationic, and anionic (Mg^2+^, Na^+^, nitrate, chloride) salts were evaluated in FBS containing 6 μM DA (Figure S17). The DPV investigation confirmed the insensitive nature of the I_2_-CMP-CNT-4-modified GCE towards AA, L-cystine (Cyst), arginine (Argrn), Mg^2+^, Na^+^, nitrate, and chloride, which is in accordance with previous results. Likewise, stability and reproducibility play a decisive role in the detection of DA in practical applications. The stability of the prepared probe was analyzed by CV by repeating the cycle at a fixed concentration of DA (4 μM) at a scan rate of 100 mV/s. Upon deducing the data, it was found that the obtained CV result had a detained current response (Figure S18). From the data, we calculated the mean, standard deviation (SD), and relative standard deviation (RSD) as 91 ± 2 with RSD of 2.8%, respectively (*n* = 8 days). Similarly, the shelf life and reproducibility of the modified GCE were tested by storing the electrode at room temperature and measuring the DPV response on alternate days. For a clear study, we separately cast the electrode for 5 and 6 μM DA concentrations. From the data, the peak current is stable, in accordance with the previous results. Additionally, we calculated the mean, standard deviation (SD), and relative standard deviation (RSD) as 44 ± 2%, and 50 ± 2% with RSD of 2.69% and 2.22% for 5 and 6 μM DA, respectively (*n* = 8 days) (Figures S19 and S20). In brief, the nanocomposite-modified GCE exhibited excellent selectivity and sensitivity for the determination of DA, along with high reproducibility and stability. In addition, the aromatic framework of CMP induces π-π stacking interactions towards the phenyl moiety of DA and boosts the selective sensing of DA from interferents [48,49,50].

## 4. Conclusions

In this study, the CNT-embedded, weak-crystalline, two-dimensional conjugated microporous poly(arylene-ethynylene)-4 was synthesized using a one-pot solvothermal synthesis. The electrical conductivity of the COF was enhanced by doping I_2_ onto the CMP-CNT-4 surface. This nanocomposite demonstrated a large increase in the anodic peak current, signifying that the as-synthesized I_2_-CMP-CNT-4 could be employed as an electrochemical sensor for the fast detection of DA. The composite provides enhanced support for the adsorption of DA and increases the mobility of electrons between the GCE and analyte, which creates a remarkable increase in the electron transfer rate compared to GCE. Moreover, this is a simple and low-cost method for the one-pot synthesis of the CMP-CNT-4-based probe for DA sensing. In addition, the prepared probe has acceptable selectivity, outstanding sensitivity, stability, reproducibility, and detection limits of 1 and 1.7 μM based on cyclic voltammetry (CV) and differential pulse voltammetry (DPV), respectively. Moreover, the current study is based on the perspective of chemistry and focuses on two areas: (1) Boosting the electrical conductivity of COF through increasing the crystallinity and incorporating charge carriers. (2) The charge transport mechanism in I_2_-CMP-CNT-4. In addition, this article provides an idea regarding the alteration of an amorphous covalent network into a crystalline structure. This crystalline modification in the network enhanced the unidirectional charge mobility.

## Figures and Tables

**Table 1:** A comparison study of I_2_-CMP-CNT-4-modified GCE with other modified GCEs.

Modified Electrode	The Detection Limit (μM)	Detection Method	References
Au/Pt/GCE	24	DPV	[47]
GCE/Au-Cu_2_O/rGO	3.9	DPV	[51]
PDA-RGO/Au	3.2	LSV	[52]
PyC	2.3	CV, DPV	[53]
GC/SDSLDH/AuNP	Not given	DPV	[54]
Pdots@AMP-Cu	4	Fluorescence	[55]
WS_2_ QDs	3.3	Fluorescence	[56]
Ag@MPBA-ABCDE	5.9	Fluorescence	[57]
Ag NP	6	Colorimetric	[58]
I_2_-CMP-CNT-4	1, 1.7	CV, DPV	This work

## Supplementary Materials

The following supporting information can be downloaded at: https://www.mdpi.com/article/10.3390/bios13030308/s1, Figure S1: Reactants and synthesis method for CMP-4. Figure S2 (a): Brown colored CMP-4, (b) Soxhlet method purification of CMP-CNT-4 in methanol, (c) Black colored I2-CMP-CNT-4 and brown colored CMP-CNT-4, (d) I2-CMP-CNT-4 modified GCE, (e) Fluorescence from CMP-4. Figure S3: SEM image of CMP-CNT-4. Figure S4 (a): The TEM image shows the average size of CMP-4, (b): SEM image of CMP-CNT. Figure S5: Solid state 1H NMR spectrum of poly(arylene-ethynylene), a chemical shift in ppm. Figure S6: Raman plot for CMP-4 and CNT. Figure S7: (a) BET surface area plot of CMP-CNT-4, (b) Isotherm adsorption-desorption plot for CMP-CNT-4, (c) Isotherm pressure composition plot for CMP-CNT-4. Figure S8: Electrochemical redox reaction in DA. Figure S9 (a) Scan rate (4 μM) from 50–500 mV/s, (b) current vs scan rate, (c) current vs (scan rate)1/2. Figure S10: log(V) Vs log(A) plot 4 μM DA in PBS (7.4 pH). Figure S11: Nyquist plot for Bare GCE, CMP-4 modified GCE, CMP-CNT-4 modified GCE and I2-CMP-CNT-4 modified GCE. Figure S12: DPV plot for DA in FBS and linear regression equation from 8–18 μM DA. Figure S13: DPV plot of 1 μM AA in PBS (7.4 pH). Figure S14 (a): CV plot for the interference of anions, (b–e) DPV plot for the interference of anions. Figure S15: Interference DPV plot of DA, L-Cyst & GA. Figure S16: Interference DPV plot of DA in Ca2+, K+ &Na+. Figure S17: Interference plot in FBS medium for 6 μM DA in presence of AA, amino acids, cations, and anions. Figure S18 (a): Current VS. potential CV plot of 5 μM DA (7.4 pH) in 20 cycles for stability studies, (b) Current Vs. cycle plot of I2-CMP-CNT-4 modified GCE in 20 cycles. Figure S19: Peak reproducibility DPV plot of 5 μM DA (7.4 pH) in 8 days, and Current Vs. day plot and RSD value. Figure S20: Peak reproducibility DPV plot of 6 μM DA (7.4 pH) in 8 days, and current Vs. day plot and RSD value. Figure S21: CV plot for the electrochemical behavior of CNT-modified GCE towards DA. Figure S22: Stability study of I2-CMP-CNT-4 (1 μM DA) at various time duration in PBS (7.4 pH). Figure S23: C(1s) deconvolution spectra of CMP-CNT-4.
