# Supplementary material for: Framing of Poly(arylene-ethynylene) around Carbon Nanotubes and Iodine Doping for the Electrochemical Detection of Dopamine
Source: Biosensors (Basel). 2023 Feb 22;13(3):308. doi: 10.3390/bios13030308 (PMC10046453; doi:10.3390/bios13030308)
Supplement: Supplementary file 1 [file biosensors-13-00308-s001.zip › biosensors-2180805-supplementary.pdf]

Electronic Supplementary Information

for

Framing of poly(arylene-ethynylene) around carbon nanotubes and iodine doping for the electrochemical detection of dopamine

Jose Paul, Md Moniruzzaman*, and Jongsung Kim*

Department of Chemical and Biological Engineering, Gachon University, 1342 Seongnam-daero, Seongnam-si, Gyeonggi-do 13120, Republic of Korea

Section A. synthesis procedure and experimental plots

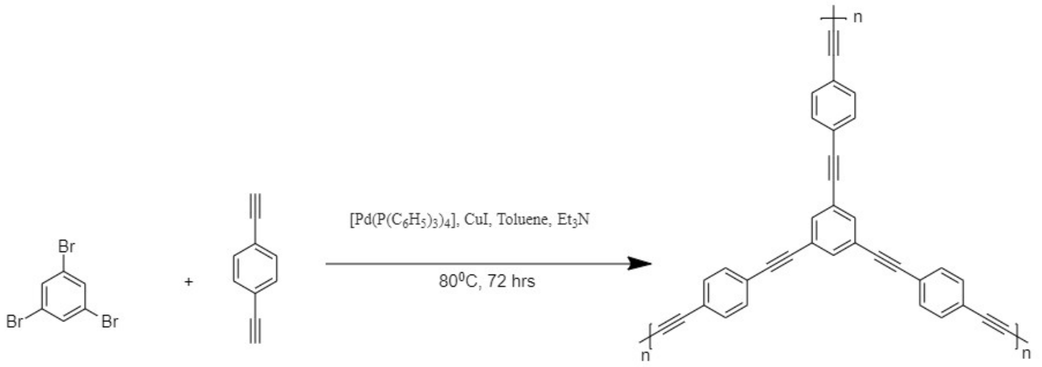

Figure S1. Reactants and synthesis method for CMP-4.

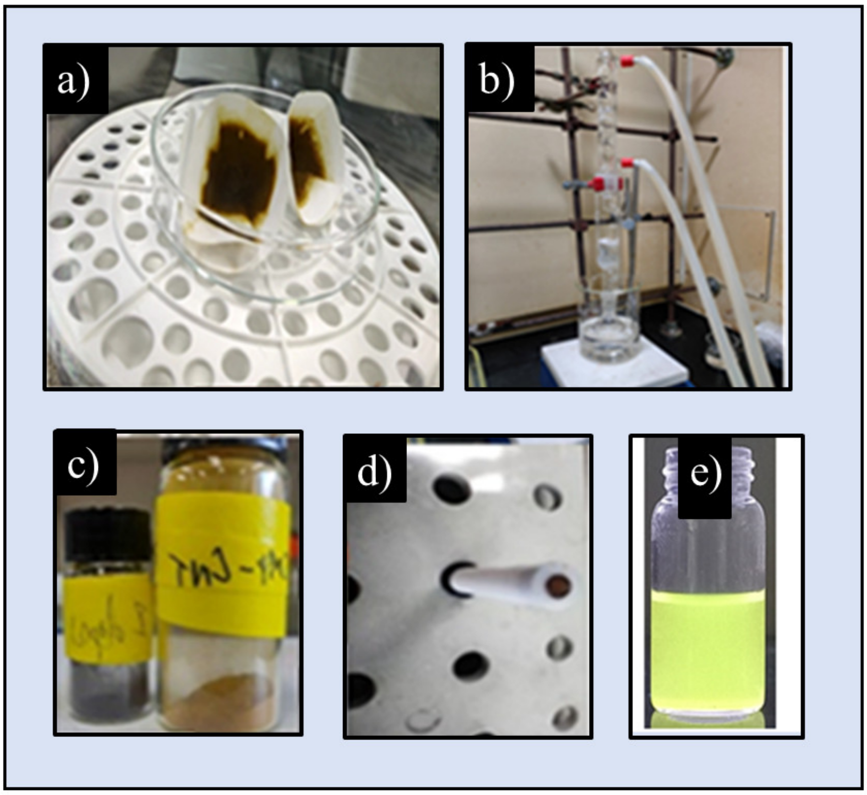

Figure S2. (a): Brown colored CMP-4, **(b)** Soxhlet method purification of CMP-CNT-4 in methanol, **(c)** Black colored I_2-CMP-CNT-4 and brown colored CMP-CNT-4, **(d)** I_2-CMP-CNT-4 modified GCE, **(e)** Fluorescence from CMP-4.

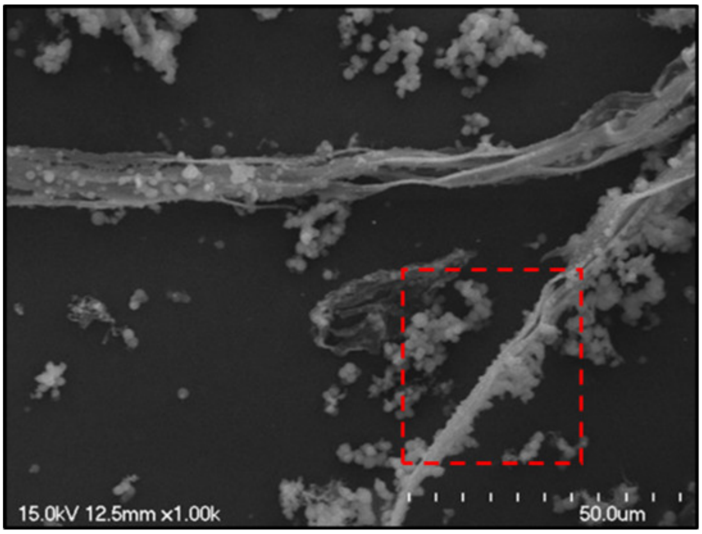

Figure S3. SEM image of CMP-CNT-4.

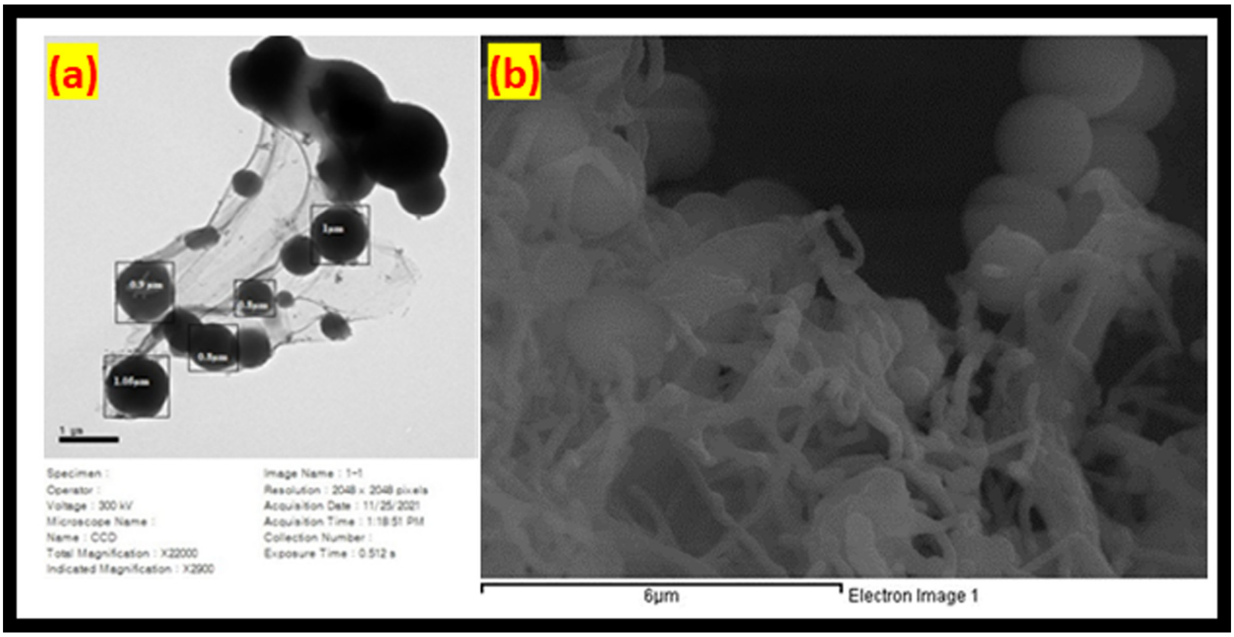

Figure S4. a: The TEM image shows the average size of CMP-4, **b:** SEM image of CMP-CNT

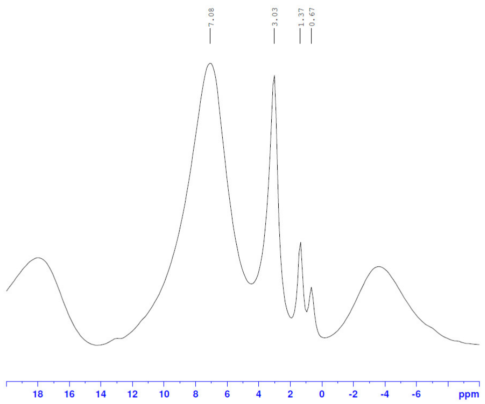

Figure S5. Solid state ^{1}H NMR spectrum of poly(arylene-ethynylene), a chemical shift in ppm.

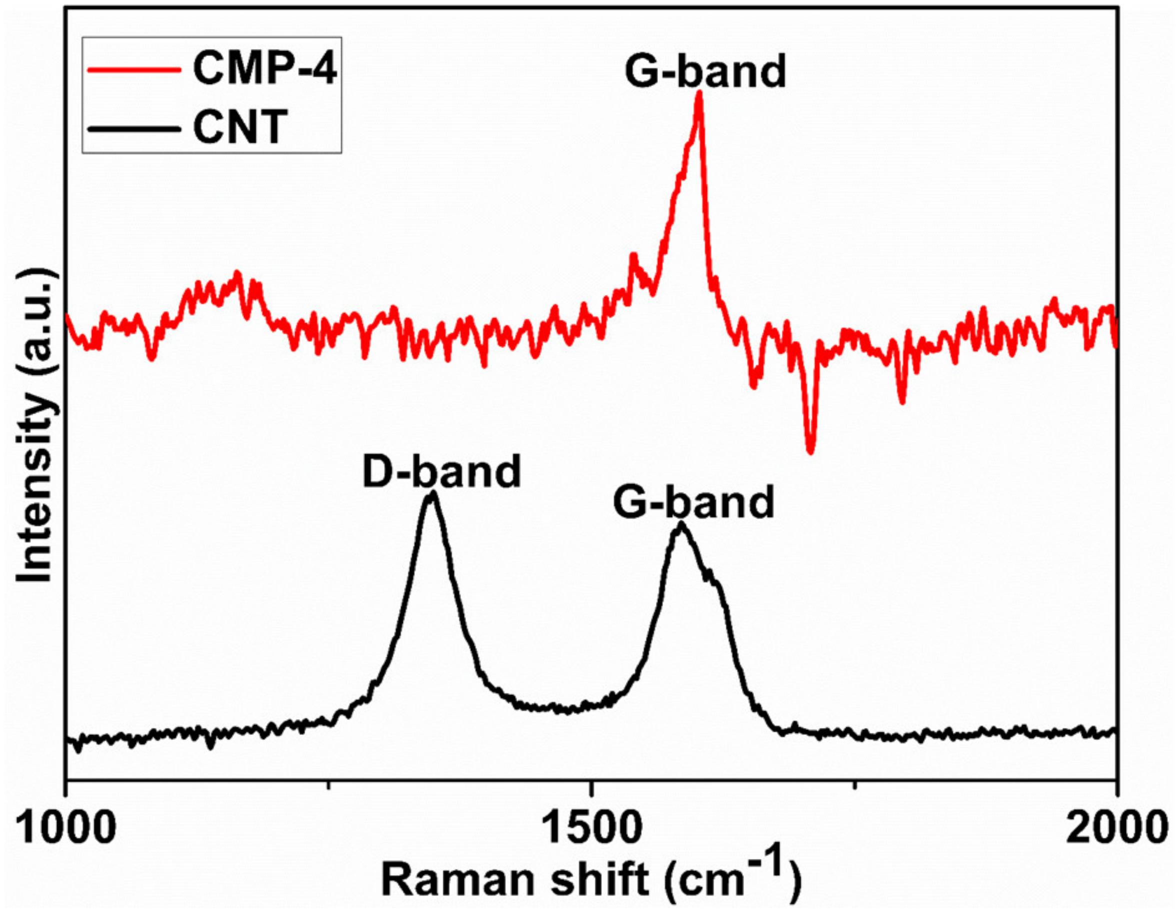

Figure S6. Raman plot for CMP-4 and CNT.

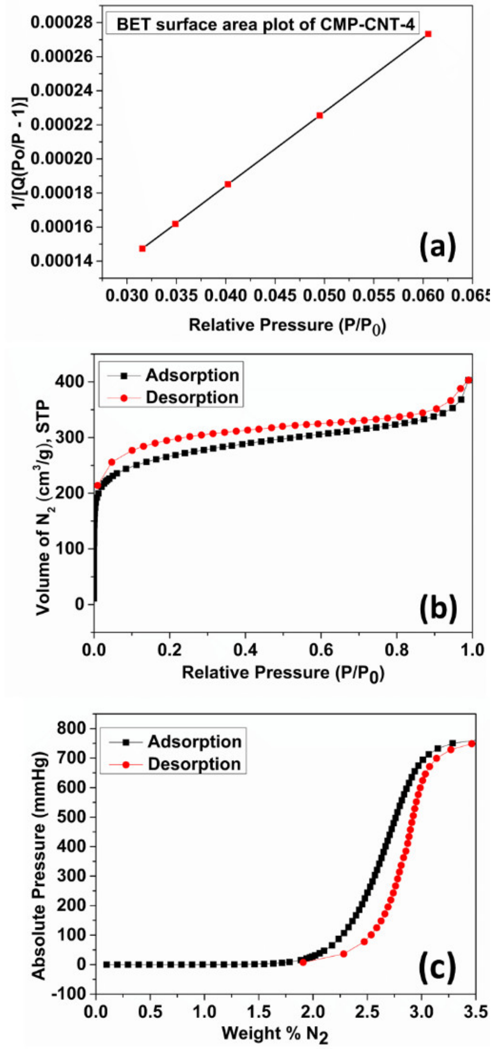

Figure S7. (a) BET surface area plot of CMP-CNT-4, **(b)** Isotherm adsorption-desorption plot for CMP-CNT-4, **(c)** Isotherm pressure composition plot for CMP-CNT-4.

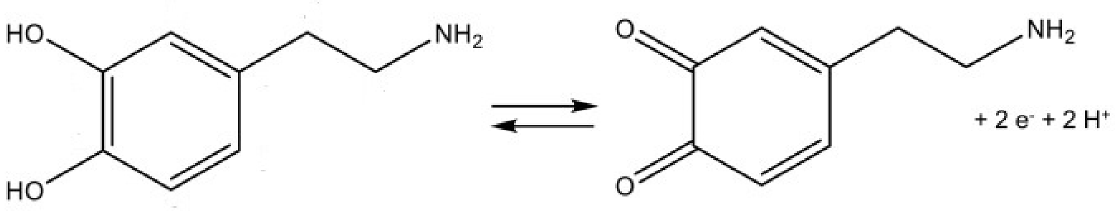

Figure S8. Electrochemical redox reaction in DA.

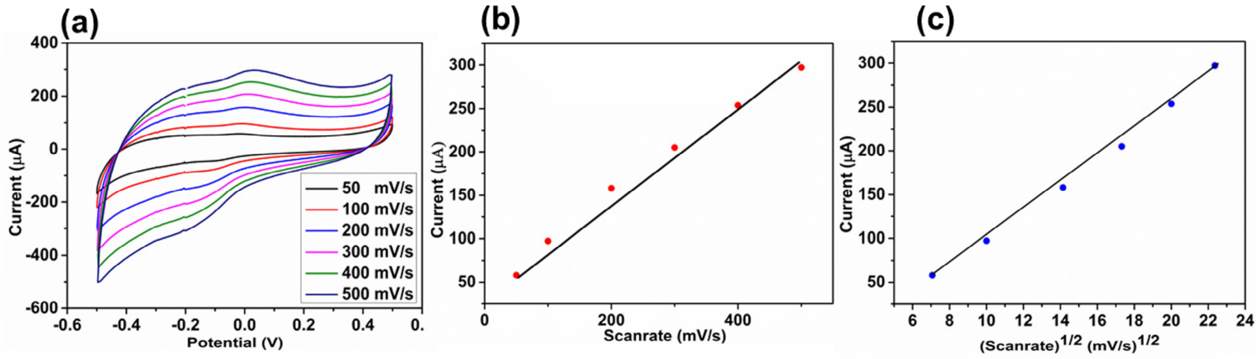

Figure S9. a) Scan rate (4μM) from 50 – 500 mV/s, **b)** current vs scan rate, **c)** current vs (scan rate)$^{1/2}$.

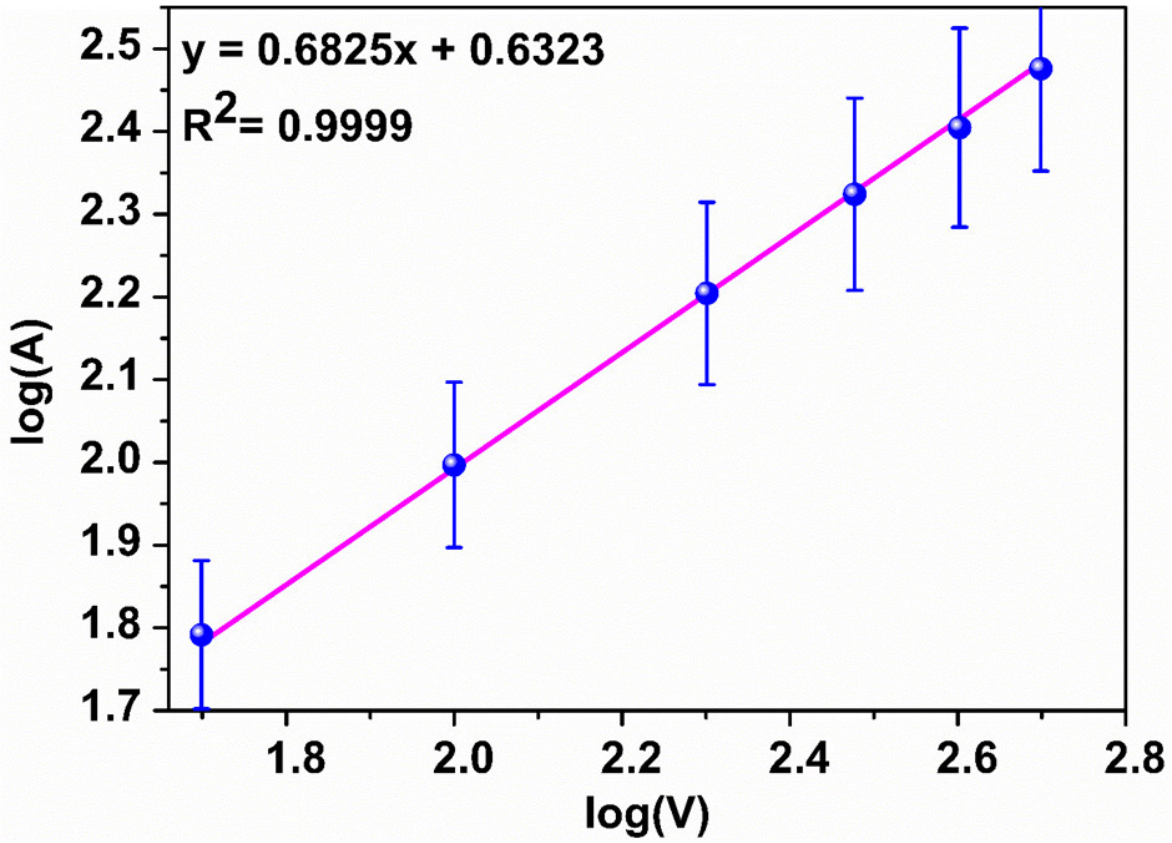

Figure S10. log(V) Vs log(A) plot 4μM DA in PBS (7.4 pH).

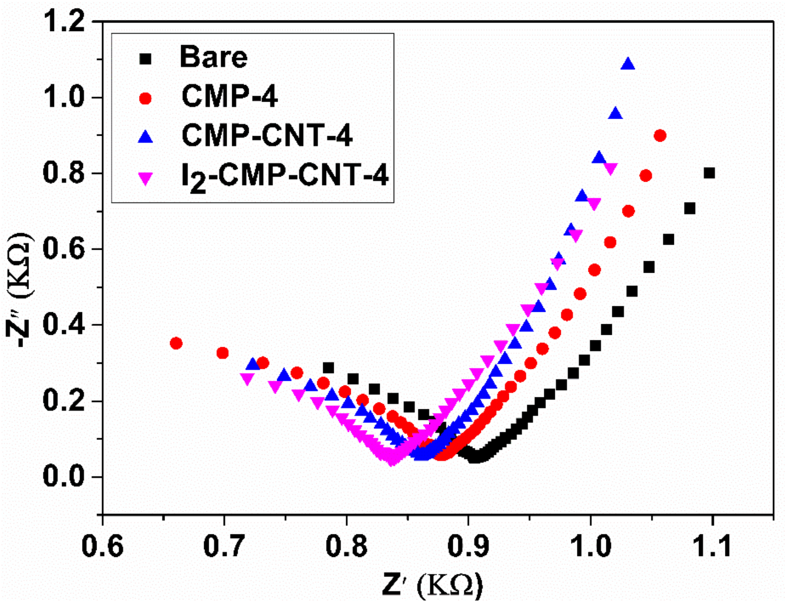

Figure S11. Nyquist plot for Bare GCE, CMP-4 modified GCE, CMP-CNT-4 modified GCE and I_2-CMP-CNT-4 modified GCE.

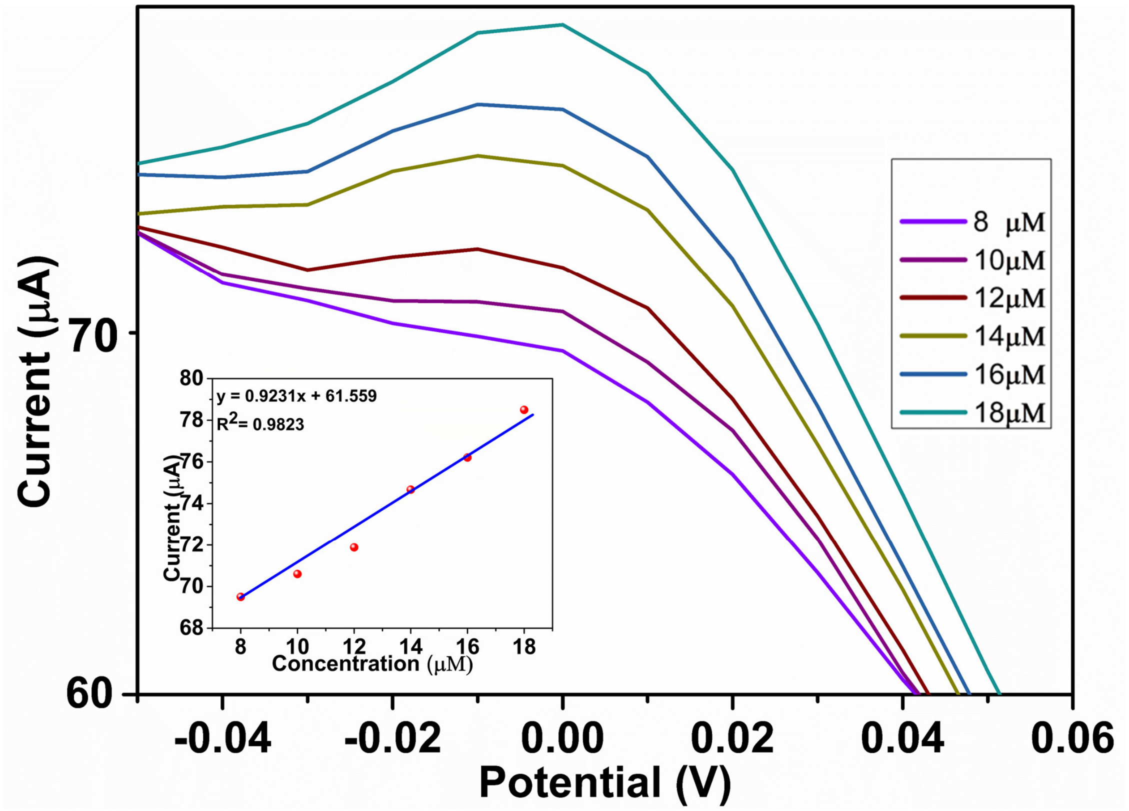

Figure S12. DPV plot for DA in FBS and linear regression equation from 8- 18 μM DA.

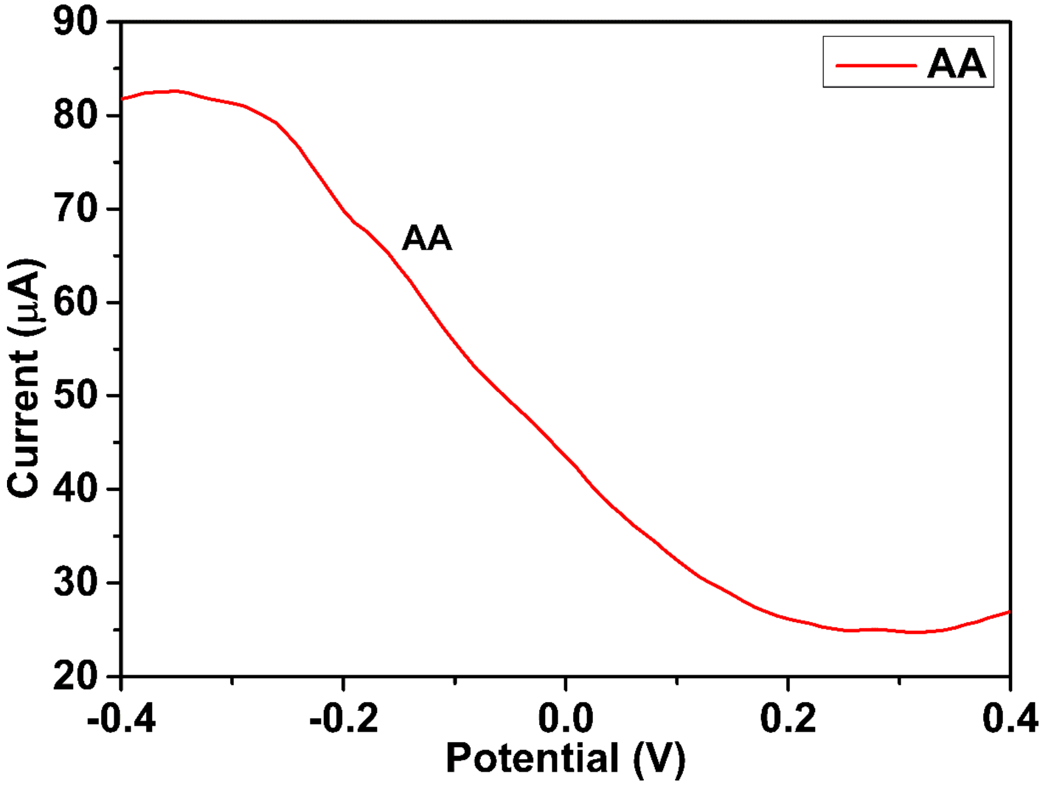

Figure S13. DPV plot of 1μM AA in PBS (7.4 pH).

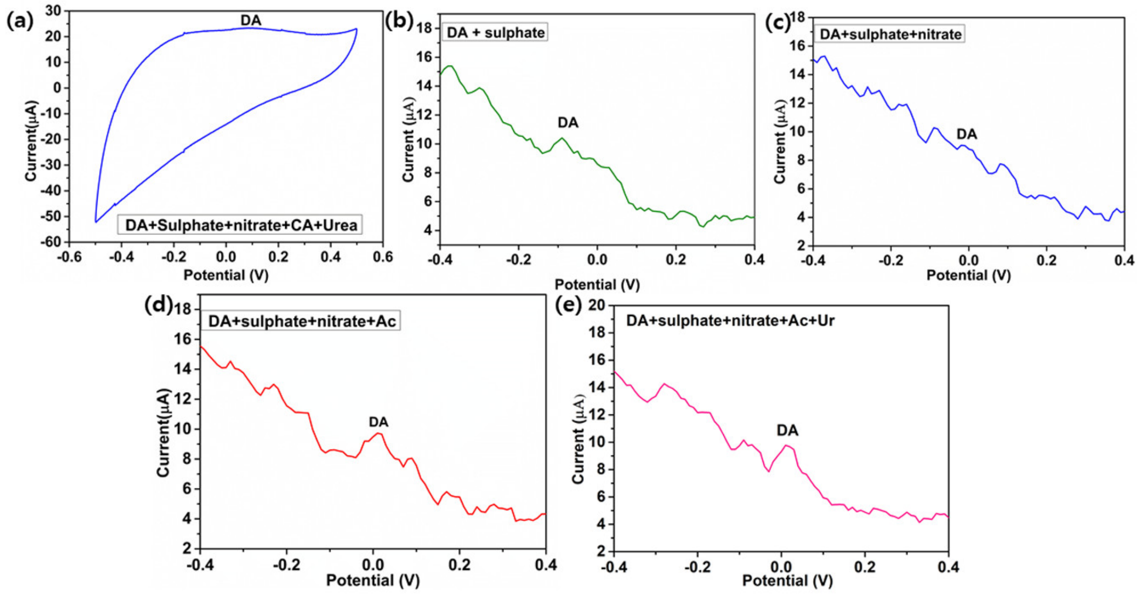

Figure S14. (a) CV plot for the interference of anions, **(b-e)** DPV plot for the interference of anions.

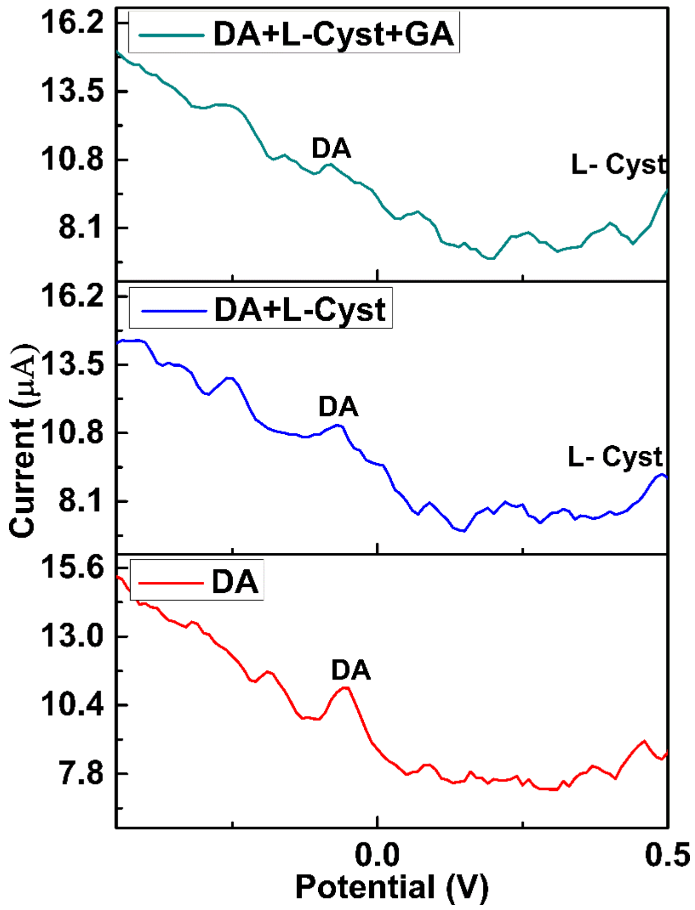

Figure S15. Interference DPV plot of DA, L-Cyst & GA.

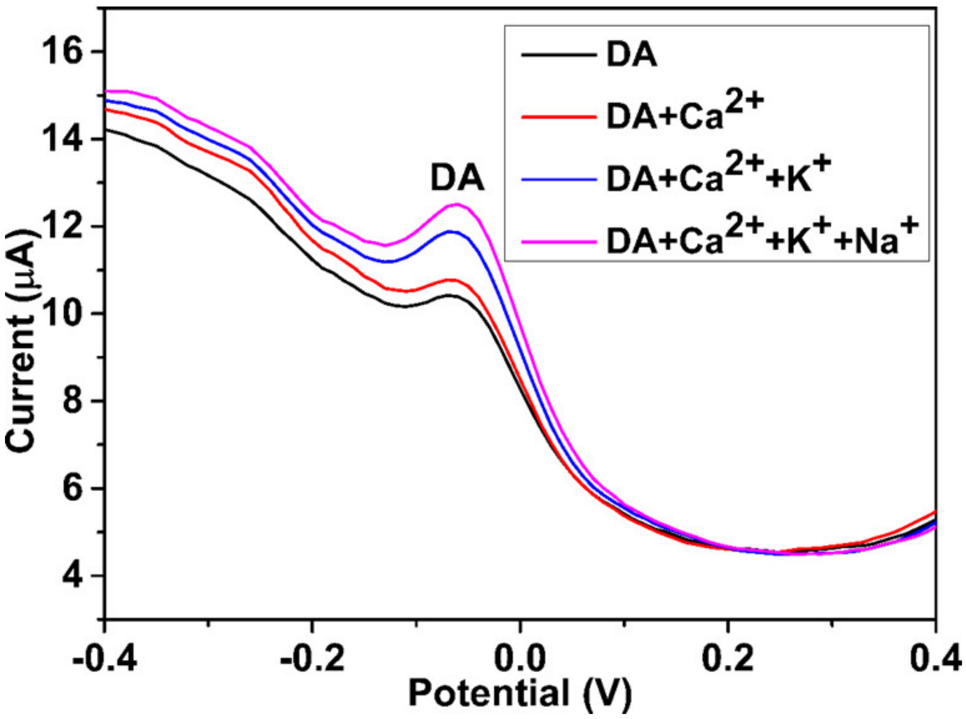

Figure S16. Interference DPV plot of DA in Ca^{2+}, K^{+} &Na^{+}.

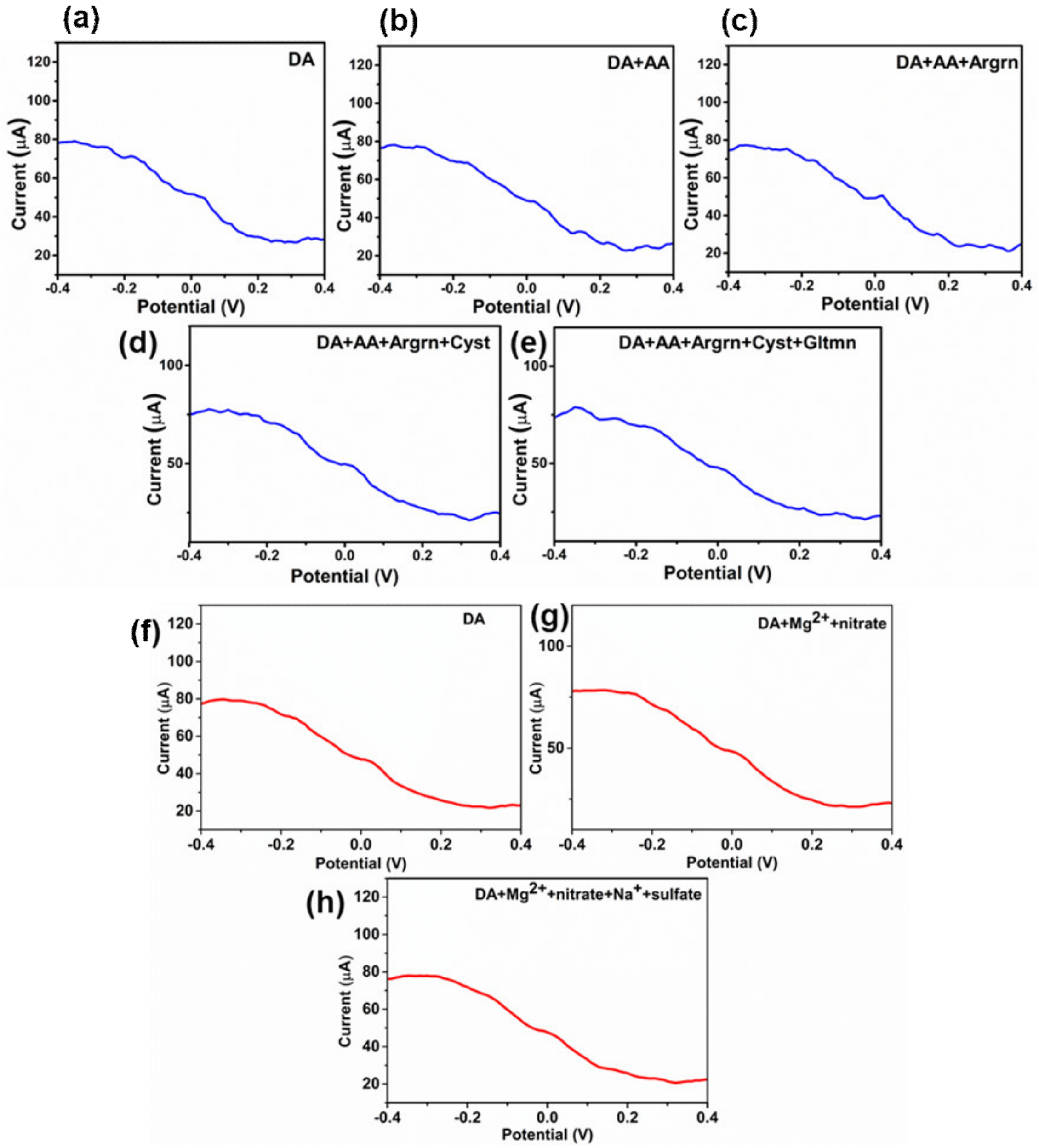

Figure S17. Interference plot in FBS medium for 6 μM DA in presence of AA, amino acids, cations, and anions.

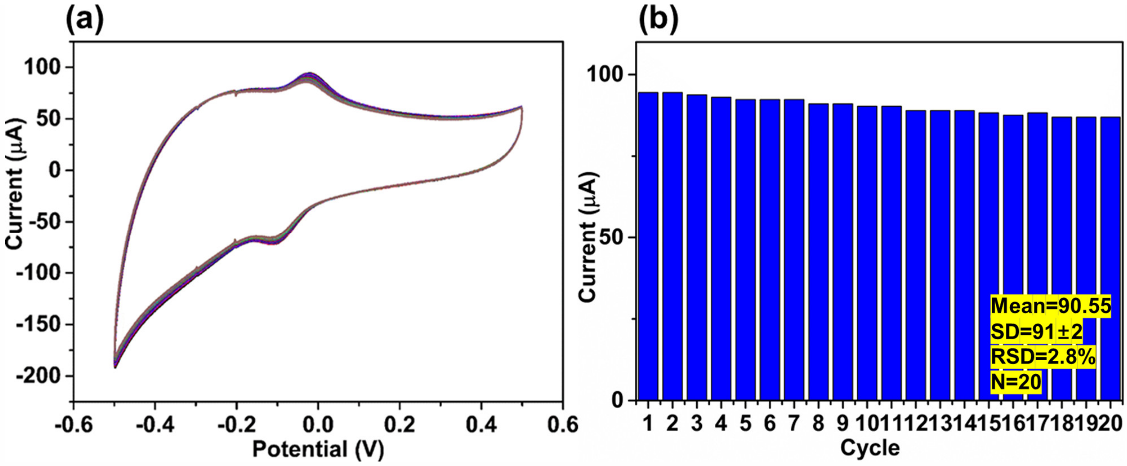

Figure S18. a) Current VS. potential CV plot of 5μM DA (7.4 pH) in 20 cycles for stability studies, **b)** Current Vs. cycle plot of I_2-CMP-CNT-4 modified GCE in 20 cycles.

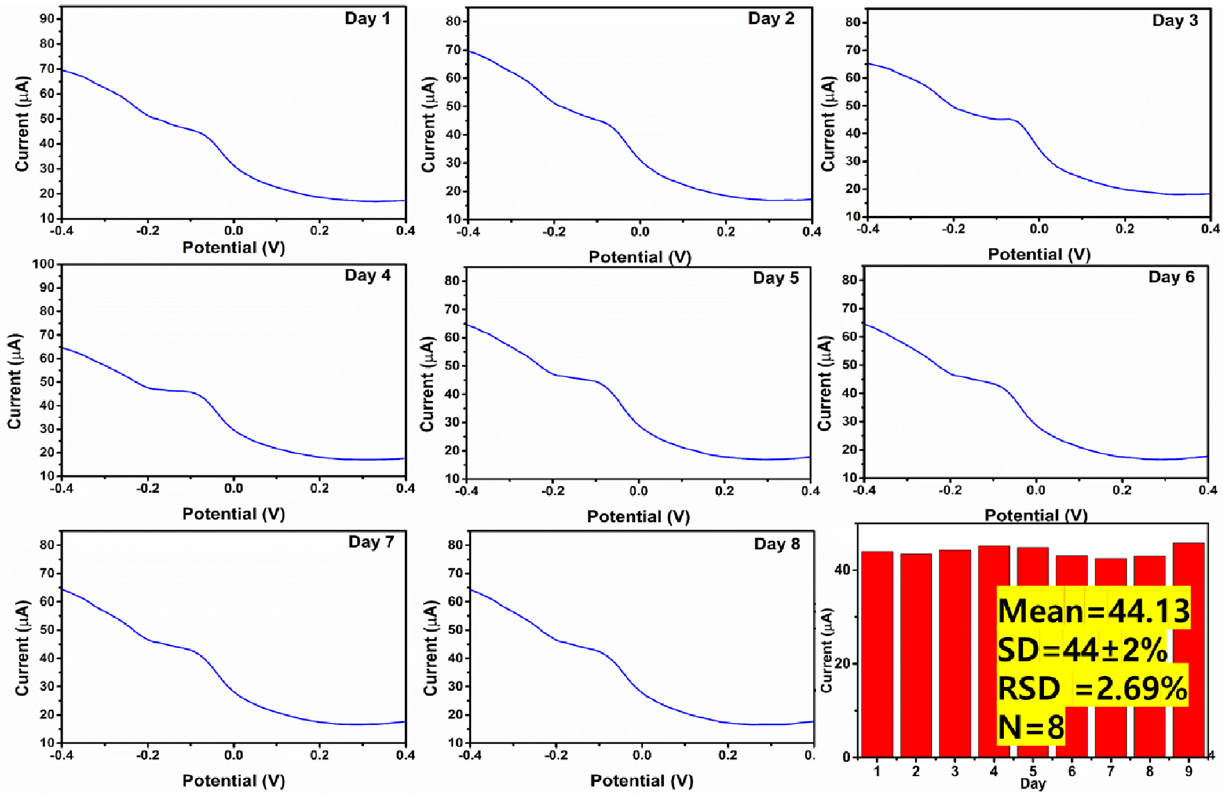

Figure S19. Peak reproducibility DPV plot of 5μM DA (7.4 pH) in 8 days, and Current Vs. day plot and RSD value.

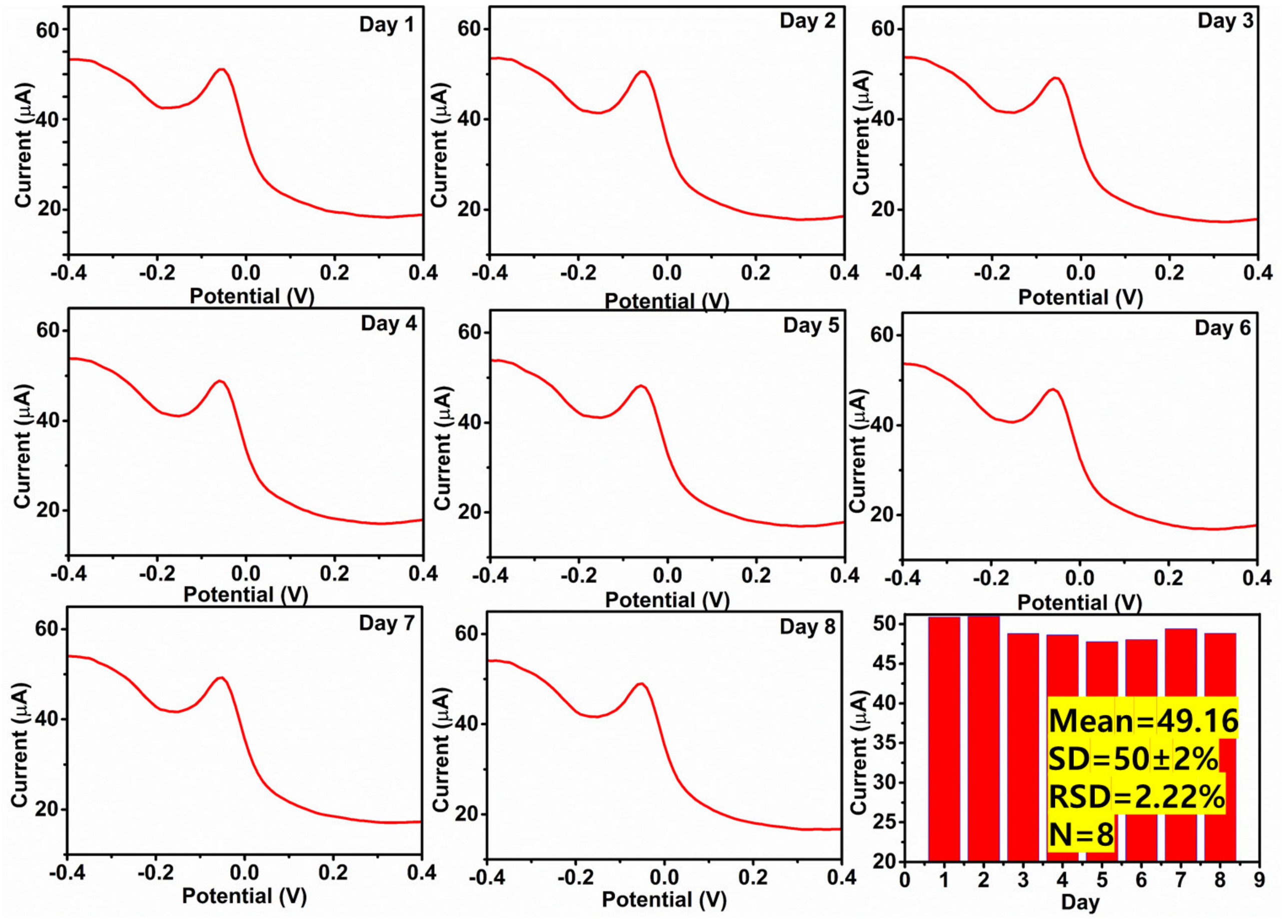

Figure S20. Peak reproducibility DPV plot of 6μM DA (7.4 pH) in 8 days, and current Vs. day plot and RSD value.

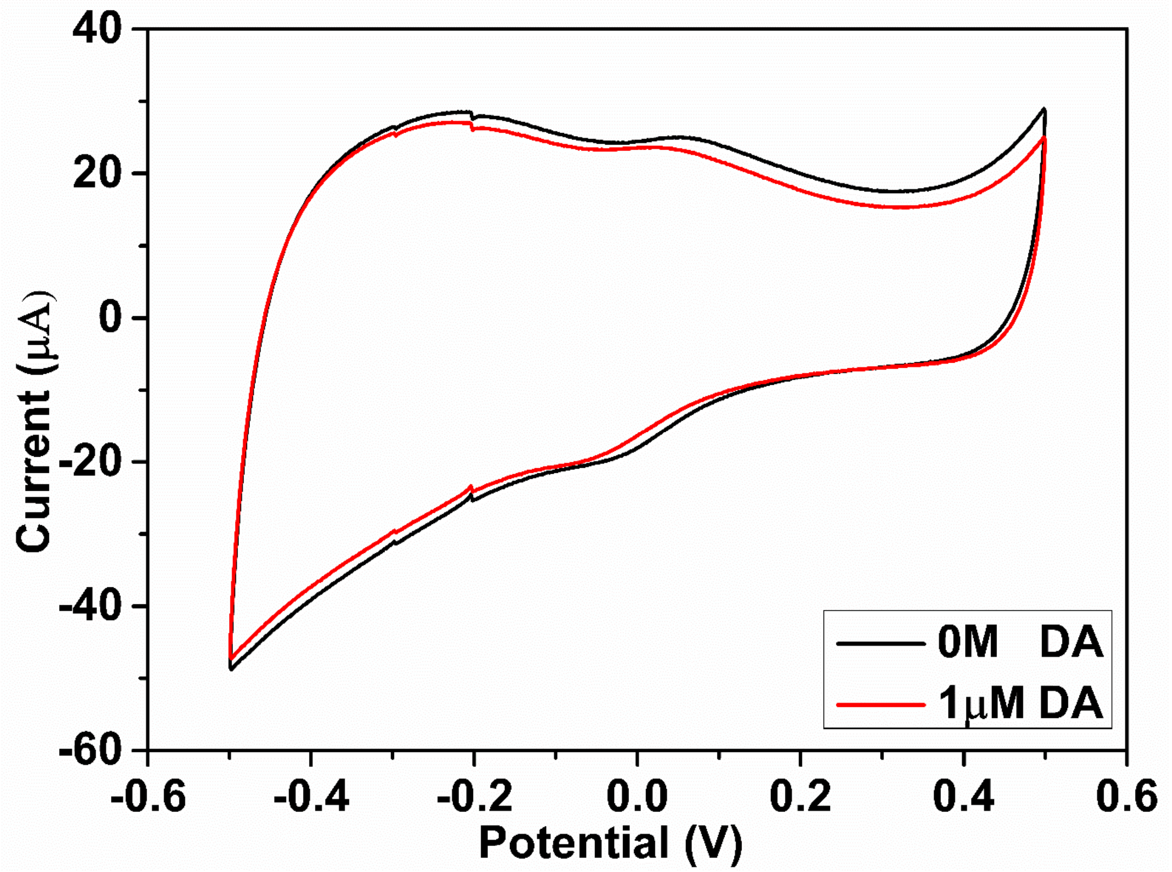

Figure S21. CV plot for the electrochemical behavior of CNT-modified GCE towards DA.

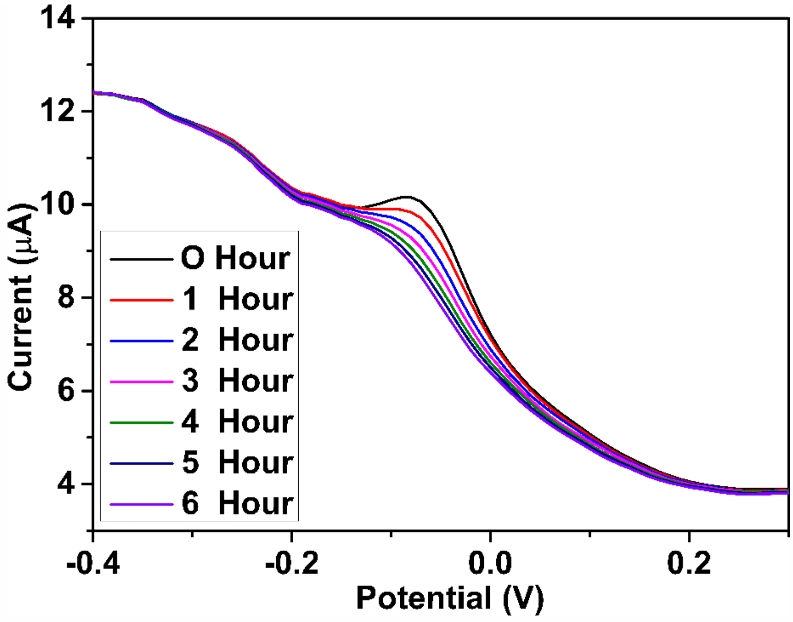

Figure S22. Stability study of I_2-CMP-CNT-4 (1 μM DA) at various time duration in PBS (7.4 pH).

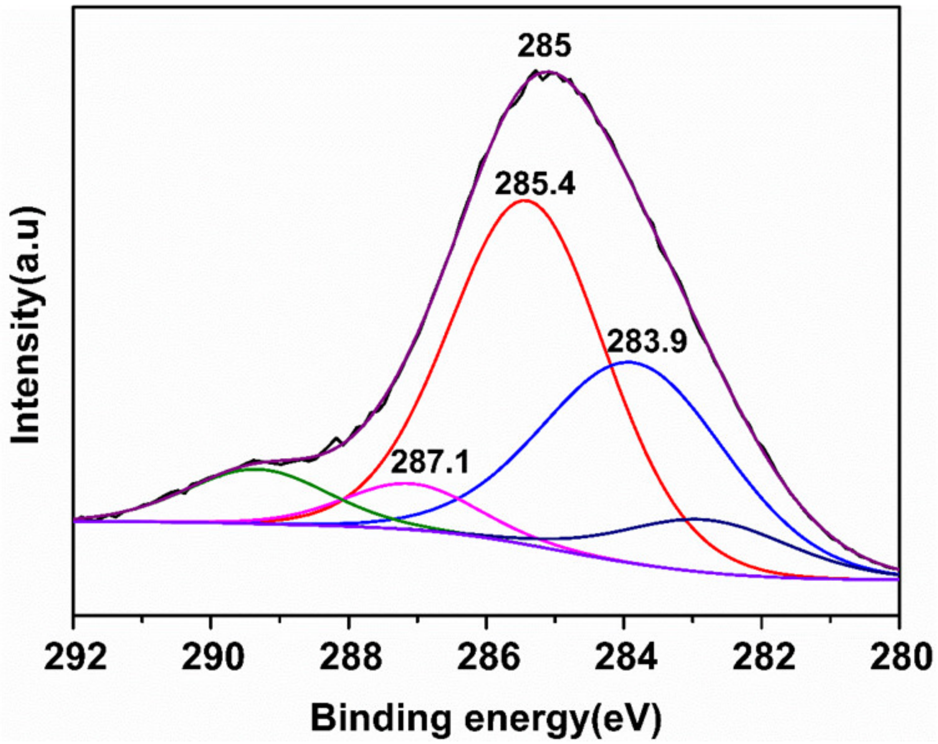

Figure S23. C(1s) deconvolution spectra of CMP-CNT-4.
